# Primary Health Care: a strategic framework for the prevention and control of chronic non-communicable disease

**DOI:** 10.3402/gha.v7.24504

**Published:** 2014-08-04

**Authors:** Alessandro R. Demaio, Karoline Kragelund Nielsen, Britt Pinkowski Tersbøl, Per Kallestrup, Dan W. Meyrowitsch

**Affiliations:** 1 Copenhagen School of Global Health, University of Copenhagen, Copenhagen, Denmark; 2 Harvard Global Equity Initiative, Harvard Medical School, Boston, MA, USA; 3 World Diabetes Foundation, Gentofte, Denmark; 4 Department of International Health, Immunology and Microbiology, University of Copenhagen, Copenhagen, Denmark; 5 Department of Public Health, Center for Global Health (GloHAU), Aarhus University, Aarhus, Denmark; 6 Health Services Research, Department of Public Health, University of Copenhagen, Copenhagen, Denmark

**Keywords:** PHC, NCDs, LMICs, integrated approach, health policies, health promotion

## Abstract

In 2014, chronic, non-communicable diseases (NCDs) represent the leading causes of global mortality and disability. Government-level concern, and resulting policy changes, are manifesting. However, there continues to be a paucity of guiding frameworks for legislative measures. The surge of NCDs will require strong and effective governance responses, particularly in low and middle-income countries. Simultaneously following the 2008 World Health Report, there has recently been renewed interest in Primary Health Care (PHC) and its core principles. With this, has come strengthened support for revitalizing this approach, which aims for equitable and cost-effective population-health attainment. In this light and reflecting recent major global reports, declarations and events, we propose and critique a PHC approach to NCDs, highlighting PHC, with its core themes, as a valuable guiding framework for health promotion and policy addressing this group of diseases.

Chronic, non-communicable diseases (NCDs) already pose an enormous risk to global health, as well as social and economic development (1, 2). Defined by the World Health Organization (WHO) as cardiovascular diseases, cancers, diabetes and chronic respiratory diseases, NCDs represent a varied group of largely preventable illnesses with complex social, economic and environmental aetiologies (3, 4). Posing many unique and diverse public health challenges, concern for the rapidly rising burden of NCDs is increasing. The added complication of multi-morbidity which is the norm in chronic conditions underlines the urgency of comprehensive approaches (5).

This was reflected in the 2011 United Nations High Level Meeting (HLM) in New York and later followed up by the WHO Global Action Plan for the Prevention and Control of Non-communicable Diseases 2013–2020 (6). As a consequence and in recognition of the crosscutting importance, a United Nations Inter-Agency Task Force on the Prevention and Control of Non-communicable Diseases has been established (7).

As yet though, there is no clear framework or guiding principles, despite many callings for a holistic approach to health promotion and action, which reflects strong social determinants (8–10). It is also widely argued that responses must reach beyond the health system into related, causative sectors (11) and there are currently efforts to advocate for appropriate technologies and cost-effective population-level interventions (9). Despite this, no single frame of principles is available.

Simultaneously, there has been renewed interest in Primary Health Care (PHC) in recent years following the 2008 World Health Report. Originally formalized at the International Conference on PHC held in Alma Ata, PHC (Box 1) has seen limited application at the government level (12), despite examples of successful community-level implementation (13).

***Box 1*. A Summary of the Alma-Ata Declaration, Primary Health Care approach.**

Primary Health Care Approach (15)

- Integrated approaches including referral of care and information as required:
  Should be sustained by integrated, functional, and mutually supportive referral systems, leading to progressive improvement of comprehensive health care for all
- Emphasizing the importance of community participation:
*Requires and promotes maximum community and individual self-reliance and participation*
- Inter-sectoral focus and private-sector involvement:
*Involves, in addition to health sector, all related sectors and aspects of national and community development*
- Focusing on equity in healthcare and in health; and reflecting the needs of the community:
*Addresses main health problems in the community*
- Cost-effective, evidence-based and affordable solutions, including community health workers:
*Reflects and evolves from economic conditions and sociocultural and political characteristics of the country and its communities, and is based on application of relevant results of social, biomedical, and health-services research and public-health experience*

In this light and reflecting recent major global reports (6, 9, 13, 14), declarations (11) and the 2011 United Nations HLM, this paper explores the relevance of the previously community-focused PHC approach as a population-level framework to guide global chronic condition-related health promotion and policy.

## Strengths of a PHC approach to prevention and control of NCDs

PHC as an approach and philosophy is centered on a number of core principles. Originally providing guidance for predominantly communicable disease prevention, many of these facets are well suited as a guiding framework for developing policy for NCDs. This paper focuses on five summarized elements of PHC included in Box 1, above:

### PHC encourages an integrated approach to healthcare and prevention

Healthcare strategies for NCDs should reflect the complex nature of the diseases, including co-morbidities. PHC places an emphasis on strengthening healthcare systems at all levels, but particularly at the primary care level, promoting not only the notion that healthcare is delivered in an integrated fashion but also that primary care is prioritised and valued by governments (16, 17). This is particularly crucial in the fight against chronic, NCDs, for which a focus must be primary, and to a lesser degree, secondary prevention and the most appropriate stage for such prevention strategies is primary care (9, 16).

In 2010, the WHO released a report outlining current evidence-based interventions for Non-communicable Diseases in primary healthcare (9). This report stressed the importance of integrating care into existing healthcare systems rather than taking a vertical approach or creating parallel programs. This approach, which reflects the core values of PHC, is increasingly reflected in interventions. An example of this is the combined treatment of both HIV and diabetes in chronic care clinics, by an international, non-government organization in Cambodia. Recognizing these two chronic diseases have common determinants and requirements from the health-care system (regular monitoring and medication, life-long follow-up, etc.), this organization capitalized on this and the strengths of an integrated approach. They also found that through a focus on chronic disease, rather than HIV or diabetes alone, community stigma was reduced and medical compliance was strong (18).

Most NCDs develop slowly over many years and exposure to risk factors accumulates throughout the life-course, often resulting in disease from the third decade of life. It is debated that diabetes risk may even be influenced by the conditions a fetus is exposed to during in-utero development (19). Therefore, addressing NCDs requires a life-course approach and integration with maternal-child health (MCH) and nutrition programs would also be wise. Another example of opportunities for service-integration is in patients with tuberculosis, who are also more likely to develop diabetes, and vice-versa. In this light, there is a case for delivering diabetes prevention and care integrated with existing infectious-disease treatment (20).

### PHC emphasizes community participation

The importance of policies, which encourage community participation in health, with regards to health promotion, is exemplified by the following.

Firstly, 80% of cancers, diabetes and heart diseases worldwide are currently preventable and prevention is cost-effective (2, 3). This group of chronic conditions is not only associated with high levels of mortality, but also long-term disability and morbidity. Primary prevention must be the focus for any strategy for these diseases and so community willingness and involvement in health promotion is essential. Policies should focus on empowering and engaging communities through health promotion strategies (21).

Three main evidence-based and cost-effective, population-level interventions currently exist for chronic conditions. These are dietary salt reduction, tobacco cessation and cardiovascular medications. In addition, good evidence exists for other lifestyle-change interventions, i.e. weight loss through combined interventions improving nutrition and promoting increased physical activity, screening programs, i.e. pap-smear for cervical cancers and changes in household practices such as indoor cooking with open fires (9). All of these require a high level of community commitment to be achieved, reflecting the impact of the community on individual's behavior through social support and norms. Community participation and empowerment will be essential to achieving progress (22).

### PHC ensures inter-sectoral collaboration and private sector involvement

Chronic conditions reflect wider social determinants such as living conditions, urban environments, access to healthy foods, and education. As such, any policy response to NCDs must involve sectors beyond health. The core PHC principle of inter-sectoral collaboration guides policy in this direction, reflecting the fact that urban planning, food production and sustainability and the involvement of the private sector in health promotion will prove essential to fight cardiovascular disease, diabetes, lung diseases and many cancers. NCDs should be seen as a challenge for all sectors and reflected in policy across sectors (Table 1). Echoing the concept of ‘Health in All Policies’, chronic conditions pose great financial, social and health threats to all communities and addressing them can be seen as a central task for many ministries and all levels of government (11).

**Table 1:** Suggested intersectoral response for key NCDs interventions

Inexpensive, Evidence-Based Interventions for NCDs (WHO)	Possible Sectors Required for Policy Effectiveness
Dietary salt-reduction	Government (Food, Agriculture, Education, Health) Private (Health, Food Industry, Agriculture)
Opportunities for exercise	Government (Sports, Education, Health, Urban Planning, Transport, Policing and law-enforcement) Private (Health, Transport)
Tobacco control	Government (Sports, Education, Health, Treasury, Customs, Youth) Private (Health, Tobacco, Hospitality and Retail Industries)
Essential medicines for secondary prevention of cardiovascular disease	Government (Trade, Health) Civil society Private (health pharmaceutical and retail industries) Professional bodies (pharmacy, medicine)

The private sector and its involvement in addressing and reducing chronic conditions has already proven important (23).

Firstly, there must be strong encouragement from consumers and their governments for voluntary self- regulation of products: how goods are produced and the way in which they are advertised. This can occur through consumer groups or government policies which support self-regulation. One example in this area is the voluntary reduction in salt content by cereal manufacturers in Australia and New Zealand (9, 24).

Maintaining a level of pragmatism, it is important that private sector responses to the surge in chronic conditions are also mandated. Legislation and regulation are needed to ensure that private sector players commit to the necessary changes required, especially as these measures may impact negatively on industry profit. Many examples of opportunities exist, including the Danish tax on saturated fat in food products, the Mexican national tax on soda and New York City's efforts to reduce soda serving sizes (25–27). Another example, is the Framework Convention on Tobacco Control (23). A success for the WHO and its member nations, this convention (adopted and ratified by more than 170 nations) has shown the potential benefits of strong government leadership and demonstrates the importance of intervention in markets, which profit from products harming public health. Analogous policy frameworks could be developed for the food and alcohol industries.

### A focus on equity

These conditions affect all populations in all nations, but disproportionately burden poorer populations. Poverty is a risk factor for the development of NCDs, it is linked to other risk factors including smoking and alcohol use, and chronic conditions themselves hinder economic development and prosperity (3). In this sense, NCDs entrench and perpetuate poverty.

Therefore, ensuring horizontal equity in healthcare systems is essential in addressing this group of illnesses. This represents not only an ethical imperative, but also an opportunity for catalysing economic development and therefore policies must actively aim to address inequities in health access and focus resources on those most in need (1). An ‘equal health access for all’ philosophy, central to PHC, encourages early and regular health access by the poor, the elderly and the ill – those most affected by NCDs – and discourages regressive health policies (12, 28).

The concept of vertical equity, across levels of income and economic development, is also a crucial responsibility for policy. At international and national levels, the greatest economic and societal burden from chronic conditions is placed on poorer nations and poorest populations within nations (14). Therefore, policies that mandate the redistribution of wealth and information through foreign aid and social welfare assist countries in coping with the growing epidemic. International development agencies and global philanthropic bodies should recognize chronic conditions in their mandates and funding, thus reflecting the seriousness and scale of the problem, the interrelated nature of these diseases and the huge economic burden currently placed on low and middle-income countries (LMIC) by NCDs.

### Use of appropriate technology

Another emphasis of the 2010 WHO PEN document was the importance of low-technology, community-based care focusing on cost-effective prevention and treatment programs (9).

The Management of NCDs requires the utilization of various medical technologies, including pharmaceutical agents, medical equipment, the structure and delivery of health services and human resources. Resources for addressing NCDs are likely to be scarce and it is therefore particularly imperative that the technologies used are appropriate, that is, that they are cost-effective, affordable, safe, evidence-based and can be implemented today with proven benefit to peoples’ health (9). Whilst greater research, particularly implementation research, is required in this field, good evidence does exist for currently available, realistic population-level interventions as outlined previously.

## Challenges of a PHC approach to prevention and control of chronic conditions

Despite PHC offering a framework for guiding health promotion and management of chronic conditions, some drawbacks to the PHC approach must be recognised:

### PHC is an approach, not a solution

PHC offers a community-focused approach to health service delivery. Its principles cover a wide range of foci, which are diverse in nature. PHC should be seen as an empirical framework for policy development and implementation, not the panacea to a growing epidemic. The broad principles of the PHC approach should guide health promotion strategy, ensuring essential values and ideals are incorporated into evidence-based policy responses.

### PHC is broad and complex

It should also be recognised that it is not an ‘all or nothing’ opportunity when it comes to applying a PHC approach to policy concerning chronic conditions. The wide range of complex, condensed principles may be too complicated or costly for parallel implementation, or parts may be too technically or politically difficult to realize in the short term. In this light, the PHC framework can be seen as a set of targets for policy that can be prioritized and implemented in succession.

## Conclusion

A PHC approach to health promotion policy encourages long-term investment in prevention-focused healthcare systems and emphasizes responses, which prioritize cost-effective, primary-care-based interventions. PHC also recognizes the importance of progressive and comprehensive health policies and that addressing NCDs will require policy responses beyond the health sector (11).

PHC provides a valuable, empirical framework for addressing prevention and mitigation of chronic conditions, offering policy makers clear and important principles. PHC is not new, nor does it provide all solutions, but it is yet to be recognized for its suitability in guiding responses to the epidemic. Holistic in nature and community-focused, the core values of PHC align well with the needs of this global health threat, reflecting the epidemiology and challenges of chronic conditions and affording an approach for their mitigation responses (Table 2).

**Table 2:** PHC based policy ‘responses’ to NCDs

Challenge of NCDs	Primary Health Care Approach (15)	Possible Policy Response
Current leading cause of mortality in LMIC and worldwide	Recognizing and appropriately resourcing NCDs mitigation and prevention	Greater recognition and funding for research, prevention and mitigation with regards to NCDs
Chronic in nature with exacerbations/complications	Integrated approach to NCDs related health systems and healthcare, including across care levels (primary care to tertiary) and between health/disease programs	Policies to ensure integrated health responses
Largely preventable through community-based strategies and lifestyle change	High level of community participation in NCDs response	Focus on prevention, with special emphasis on primary care and community based programs
Highest disease burden in resource-poor settings	Cost-effective, evidence-based and affordable strategies and use of appropriate technologies (9)	An emphasis on cost-effective population-based interventions backed by evidence and global guidelines
Disproportionately affecting poor populations	A focus on equity	Policies which reflect the inequities associated with NCDs and strive to address these, recognizing NCDs are not a result of ‘choices’ but rather social inequalities and determinants
Many up-stream, social determinants	Inter-sectoral focus with NCDs related policy including non-health sectors	‘Health in all policies’ approach to NCDs including all sectors in prevention and control strategies and responses
Inter-related with communicable diseases, nutrition, maternal and child health and environmental hazards	Focus on prevention, early intervention and a whole-of-life approach	Policies which focus on social determinants of NCDs and aim to address wider causes of disease through up-stream determinants
